# Personalized Research on the Aging Face—A Narrative History

**DOI:** 10.3390/jpm14040343

**Published:** 2024-03-26

**Authors:** Marius Valeriu Hînganu, Ramona Paula Cucu, Delia Hînganu

**Affiliations:** 1 Department of Morpho-Functionall Sciences I, “Grigore T. Popa” University of Medicine and Pharmacy, 700115 Iasi, Romania; marius.hinganu@umfiasi.ro (M.V.H.); hinganu.delia@umfiasi.ro (D.H.); 2 Department of Surgery, “Grigore T. Popa” University of Medicine and Pharmacy, 700115 Iasi, Romania

**Keywords:** aging face, research history, SMAS, facial rejuvenation

## Abstract

Throughout history, people have struggled to find out the secret of youth. The aim of the manuscript is to review the main achievements regarding the exploration of the aging face phenomenon. It should be very important to know the evolution in this field due to the increase in life expectancy among the population. Our purpose is for the current study to serve as a starting point towards exploring novel research avenues in molecular biology and the confocal immunofluorescence of cervicofacial soft tissues, employing cutting-edge techniques. All changes in the shape of the facial skeleton, soft tissue, retaining ligaments, fat compartments, and the skin envelope contribute to facial aging to varying degrees.

## 1. Introduction

Throughout the millennia of history, people have struggled to find out the secret of being forever young. All along this path, they searched for magical liquors, the fountain of youth, or miraculous remedies to preserve their youth and vitality as long as possible. The known history of anti-aging treatments begins with Queen Cleopatra who, in 69 BC, bathed in donkey’s milk to maintain the quality of her skin [1].

Plato, in 400 BC, characterized aging and disease as two different phenomena. By living a healthy life, harmony can be created between the movement of the soul and the body [2].

Aristotle, Plato’s disciple, proposed that aging be considered a natural disease that comes with a natural loss of intellect [3]. ‘A disease to be hated and feared’ was the expression used to describe the same thing in 19th century.

At that time, while the phenomena of aging of the body have piqued the interest of philosophers since ancient times, the scientific community did not seem interested in deepening the subject. Aging was considered as part of the evolutionary process, and nothing more than natural selection [4]. 

The face is probably the region most rapidly associated with aging phenomena. This is due to both extrinsic factors—direct exposure to environmental factors (ultraviolet radiation, wind, humidity, or dryness), extrinsic factors (smoking, alcoholism, food habits) and intrinsic factors, which relate to the vegetative reaction to various stressors and individual genetic inheritance. All of these bring about genetic changes, acting mainly on the vascular system that serves the structures with an increased risk of aging—the superficial soft tissues of the face [5,6].

In the early stages of exploring the aging process, these findings comprise clinical observations on individuals, grounded in the examination and experimentation of potential remedies [7]. 

Intrinsic aging is determined by the passage of time (a chronological factor) and is a normal, physiological process. However, there are genetic and hormonal factors that influence this type of aging. A lot is inherited in this process, and if we look at older relatives, we can see a lot of similarities in these people, especially if they live in the same environmental conditions [8,9]. 

Hormonal factors influence the aging process in both genders, but it is known that in females, after menopause or even earlier, at 35–40 years, the process is accelerated, which proves their involvement in aging, because the level of estrogen hormones begins to decrease after this age. The disappearance of subcutaneous fat for various reasons accentuates the aging of the face [9]. 

The extrinsic aging process is easier to control because it occurs under the action of factors outside the body that we can protect ourselves from. Ultraviolet and sunlight have a negative role, accelerating the aging of the skin. Other factors are alcohol, smoking, lack of sleep, mental stress, diets, drug abuse, various diseases, and existential habits [10]. 

The influence of gravity has the same effect on youth as in old age, but tissue displacement is limited in youth (occurring only when the tissue becomes lax) [11,12].

The causes that lead to premature aging of the skin and especially of the face, are exposure to sunlight, tobacco dependence, and tissue gravitational displacement, which tends to weaken the retaining ligamentous support [10]. If a young person’s facial skin is smooth, firm, and without wrinkles, with the passage of time and under the action of the sun’s rays, signs of skin aging appear. The skin loses its elasticity, which leads to sagging and the appearance of deep wrinkles. Alterations in skin texture manifest, pores enlarge, and fine lines appear. In addition, the color of the skin changes, pigmentation spots appear, both those due to age and the sun, areas of redness (rosacea), and broken or dilated vessels appear.

The outer layer of the epidermis (stratum corneum) is the skin’s first protective barrier against the external environment. It consists of corneocytes, which are anucleated keratinocytes that have reached the final stage of keratinocyte differentiation. These cells prevent dehydration of the skin and are organized in the form of a brick and mortar wall, in an extracellular matrix rich in lipids [13].

The aim of the manuscript is to carry out a review of the main achievements that have taken place over time among the techniques for exploring the aging face phenomenon. We consider it very important to know the evolution in this field due to the increase in life expectancy among the population. We want the present work to be the starting point in a new and innovative research direction of the cervicofacial soft tissues through avant-garde techniques in the field of molecular biology and confocal immunofluorescence.

To achieve this, we have consulted the following international databases: Web of Science, Science Direct, PubMed, and Scopus. The keywords we used in the search are: aging face, research history, SMAS, facial rejuvenation, and antiaging. The included material consisted of 83,601 original articles, 5579 reviews, and 728 book chapters. From these we excluded the papers in other languages than English and papers unrelated to medicine. 

## 2. The Beginning (from Renaissance to 1900’s)

In 1550, after a long observational study, Luigi Cornaro wrote the book “The Art of Living Long” [14]. The book has become the bible of longevity and advocates that individuals should live carefully and with a good constitution. In his opinion, this is based on leading a simple life, on the principle of moderation in all things [14]. 

In another observational study, Benjamin Rush, in 1797, recommended that for a happy old age, not to overcome the laws of nature, but to understand them, so that elderly individuals remain productive members of society [15]. 

In the late 1800’s, an entire series of ‘physiological experiments’ and gland grafting techniques claimed that they discovered the ‘elixir of youth’ [16]. 

At the beginning of this period, scientists began to study the mortality rate and curves of unicellular and multicellular organisms. They promptly grasped a fundamental aspect, namely, they comprehended that most mortality curves had a similar structure. Most organisms had higher mortality rates in old age and it is not feasible to stop the aging process [17]. 

This marks the point when scientists became aware that the rate of aging could be slowed and that the same species could live drastically increased lifespans and with a superior quality of life. Therapeutic measures, including one called caloric restriction (CR), have been established and promising findings have been published [18]. CR means reducing the amount of food that an organism consumes and can lead to an increase in both lifespan and its quality [19].

The initial investigations into CR were conducted in mice, which were fed a diet that was 30 to 40 percent less than their normal caloric intake. Today it is scientifically proven that CR also prevents age-related diseases in most organisms.

Professor Michael Klass studied worms (*Caenorhabditis elegans*) to understand the genes involved in aging [20,21]. They were genetically modified using a compound called ethyl methanesulfonate and lived longer than the average, unmodified wild worm [22].

Worms that lived longer were thus found to have reduced activity in their age-1 gene, PI3-K, leading to the discovery of the insulin-like growth factor 1 (IGF-1) signalling pathway. Professor Cynthia Kenyon discovered that a single mutation in the daf-2 gene, which codes for an insulin receptor, can almost double the lifespan of *Caenorhabditis elegans* worms.

Thus, Klass’ findings paved the way for aging research. Older researchers continue to build on Klass’s studies today.

Aging of the face is manifested both in the structure of the skin and externally [23]. 

### 2.1. The External Changes of Facial Aging

An analysis of the data showed that aging in the upper part of the face (forehead and eyebrow area) and lower part (fountain and jawline) occurs more gradually, compared to the middle of the face, especially the cheek area.

The first area prone to facial fat depletion due to aging is the cheeks, immediately below the eyes, followed by the mid-cheek area. This loss in volume draws attention away from the eyes and cheeks, attributes that are considered the key features of beauty and the importance of preserving volume in these areas is crucial for sustaining a youthful appearance.

Nasolabial grooves or nasolabial folds emerge gradually, like the goiter, to the development of a generalized wrinkling of the face.

We often notice an accumulation of tissue and deep wrinkles at the corner of the mouth, and sometimes, these ‘accumulations’ interrupt the continuous line between the chin and the ears. The corners of the mouth go down, resulting in a ‘soft’ face.

As we age, we tend to lose the fine layer of fat that supports and “fills” the skin of the face, dryness sets in and skin elasticity decreases. The cheeks drop down, resulting in the protruding jaw [24].

**Figure 1:**
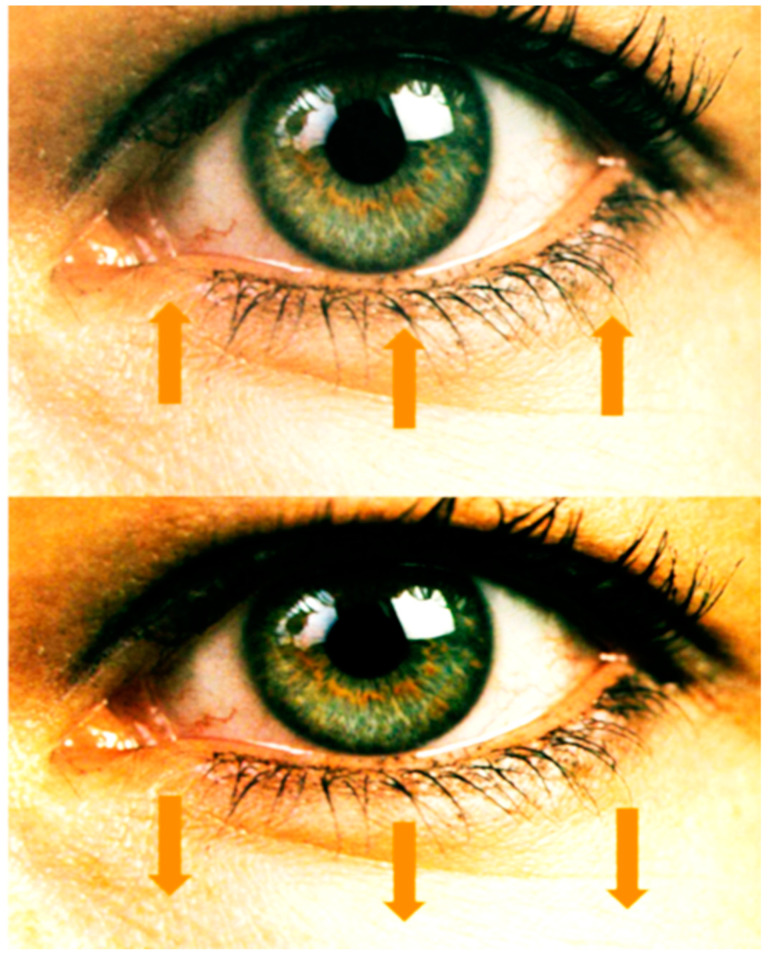
The vertical descent of the orbital region of the face due to aging and ligament laxity (personal collection).

The tissue around the eyes moves lower, the two eyelids fall. The forehead tissues fall down, drawing in the eyebrows as well, making them appear more prominent. The nose is elongated and the tip moves downwards, sometimes developing a dorsal prominence or the tip of the nose can enlarge, acquiring a “bulbous” appearance (Figure 1) [25]. 

We notice “bands” in the muscles of the neck, which generate a build-up between the chin and the neck, or a sagging of the skin of the neck. Combined with the appearance of fine wrinkles caused by the sun, these are the first signs that make us feel that we are more mature.

Vascular lesions (telangiectasias), pigmentation (hypo/hyperpigmentation), and benign or even premalignant lesions (actinic keratosis) may occur [26].

Starting between the ages of 20–25, the eyebrows descend slowly and steadily, from the level of the supraorbital arch down. This ptosis of the eyebrow, combined with an excess of skin at the level of the upper eyelid, and with the weakness of the coverings of the orbital septum, shows us the typical appearance of the aging eye. The weakness of the orbital septum and the excess laxity of the skin of the lower eyelid allows the orbital fat to herniate (resulting in the so-called palpebral bags).

**Figure 2:**
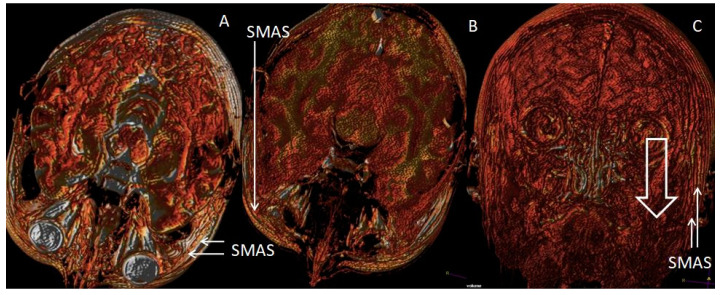
Representation of the antigravitational fixation regions of the SMAS and the retaining ligaments involved in the aging face: (**A**) Transversal 3D reconstruction on which the transverse plane of the SMAS is highlighted, corresponding to the zygomatic and temporal ligaments, from the infraorbital region to the zygoma; (**B**) Transverse 3D reconstruction highlighting the existence of the topographic plane of the SMAS insertion at the level of the nasolabial fold; (**C**) Coronal 3D reconstruction highlighting the topographic plane of the SMAS from the tragus to the gonion—the downward arrow indicates the direction of movement of the SMAS in this region in the aging face (personal collection).

A wrinkling of the skin and profound three-dimensional changes of the subjacent layers represent the substrate of the phenomenon of facial aging. These structures to which bone support is added are individually affected by this phenomenon, but they, at the same time, act as a unitary, dynamic whole that determines the phenotype of the face (Figure 2).

The young face presents a distribution of the adipose and deep layer that gives a rounded appearance to the face in all three directions of space, delimited by a series of arches and convexities [27].

Then, genetic studies emerged. The next breakthrough came in 1995, when a group of researchers in Cambridge, Massachusetts began discovering additional genes associated with longevity in the budding yeast *Saccharomyces cerevisiae*. The NAD+-dependent deacetylase SIR2, a second regulator of longevity, was discovered, taking advantage of a correlation between stress resistance and longevity. A group of researchers have identified a series of starvation-resistant mutations with an extended lifespan. One of these strains carried a mutation in SIR4 [28,29].

### 2.2. Molecular Studies Were the Next Step

From observational studies to genetic and molecular studies, it was only one step. A single step, but with an overwhelming importance, consisted of clinical studies carried out for the first time in the hospitals of Paris, by doctors which collaborated with the the advancement of the fundamental sciences in medicine—anatomy, physiology, and biochemistry. The elites of the time correlated old age with specific physiological and morphological changes that the body goes through. By quantifying tissue damage and the subsequent changes that occur in the cell, they concluded that old age was not just a decline in vitality and could be easily controlled through a regimen of diet and exercise. I. L. Nascher, the ‘father’ of geriatrics, wrote: ‘It is impossible to draw a clear line between health and disease in old age. With each organ and tissue undergoing a degenerative change that affects physiological functions, it is a matter of personal judgment to determine at what point the changes in anatomical features and physiological functions depart from the normal changes of senility and to what extent’ [30].

From the frontal incidence, the arch of the chin, the convexities of the temples, and the arches of the lips are visible (Figure 2). In profile, the cheeks, chin, and forehead protrude.

**Figure 3:**
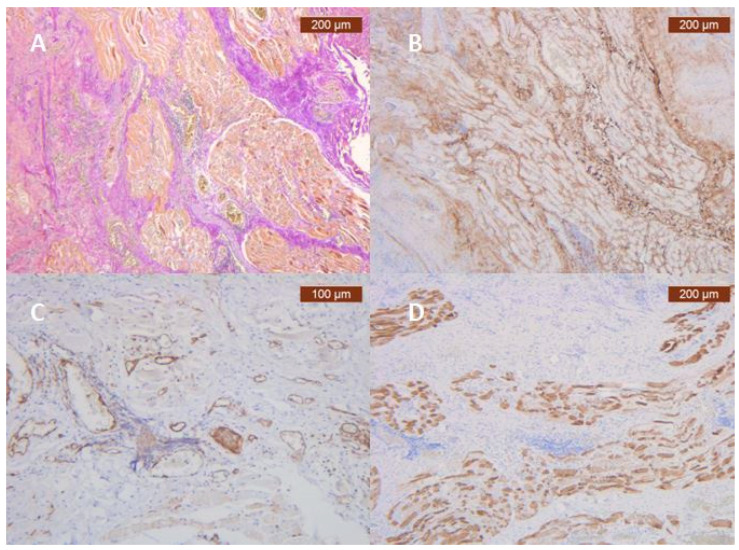
Morphohystological and special IHC techniques highlighting the existence of a proper SMAS vascular network in the infraorbital region. (**A**) Deep vertical and horizontal collagen fibrous septa (red) and muscular fibers (yellow) (VGx5, VG-vanGieson); (**B**) Abundant perivascular and perimuscular type III collagen (Anti-Collagen IIIx5). Staining intensity: moderate; (**C**) ICAM-2 is expressed on blood vessel epithelium, moderate and strong staining of the endothelium (Anti-ICAM-2 ×10). Staining intensity: weak and moderate; (**D**) Immunohistochemically strong positive myosin reaction of muscular fibers in SMAS fibrous area (Anti-MyH2 ×5). Staining intensity: moderate (personal collection).

The main role of the SMAS components is to achieve the antigravity support of the face, what we called “sustentaculum facies” [31]. The functional capabilities of this facial lift apparatus depend overwhelmingly on the integrity and regional characteristics of its vascular microperfusion. There is a direct correlation between the quality and quantity of collagen and myosin fibers in the SMAS network and the homeostasis of the blood circulation itself. Recent molecular studies demonstrate more than that, specifically the fact that SMAS has its own vascular plexiform network. Collagen type III showed diffuse fibrillar staining throughout the stroma, reproducing their characteristic fine, irregular fiber distribution and thick network. The amount and intensity were varied from moderate to strong. Immunohistochemical analysis of ICAM-2 revealed that the antibody primarily was expressed with moderate and strong intensity on the epithelium that covers blood vessels. MyH2 was present in muscle fibers in stroma. The expression in cytoplasm of spindle-striated muscular cells were more moderate to strong [32] (Figure 3).

Advancements in molecular methods have significantly contributed to our understanding of skin aging. This results from knowledge of the response to DNA damage and of different forms of stem cells depleted. Each of these contribute to development of new interventional strategies. Recent studies showed that mitochondrial DNA mutations are associated with skin aging [33].

The state-of-the-art in molecular aging biomarkers, such as telomere length, p16 INK4a expression, and epigenetic age, have emerged as valuable tools in understanding and predicting age-related changes and health outcomes. These molecular aging markers monitor various molecular alterations associated with aging. Furthermore, their collective effects result in an aging phenotype and contribute to a deeper understanding of aging processes and the development of interventions to promote healthy aging and longevity [34].

### 2.3. Morphological, Radiological, and Hystological Studies

Adipose tissue decreases quantitatively in genetically predetermined regions, such as the periorbital, frontal, zygomatic, mandibular, chin, glabellar, and perioral, and instead accumulates in larger quantities in other regions, such as the submental, nasolabial, and jugal, forming the subpalpebral fat bags and the zygomatic fat bubble [35,36]. 

The growth ratio of fat deposits in the upper part of the face compared to the middle part is 10 in the elderly and 0.1 in the young (from quantitative MRI determinations). Fat packs become gradually separated from each other, which will give the face a senile appearance [37]. 

The arches and convexities presented in young people are interrupted; the arches of the chin and mandible retreat, leaving the jugal visible, and the forehead and cheekbones also recede. The muscles of the forehead remain without an overlying tissue, which causes the appearance of wrinkles in this region. In the face regions, active expression wrinkles turn into passive wrinkles.

The aging phenomena of the face can appear and progress differently contralaterally, which leads to facial asymmetries.

Following the loss of the periorbital connective tissue, the fat at this level and the lower fascicle of the orbicularis oculi end up being covered by the lower eyelid, which gives a dark color to the skin. This gives the look of tired eyes.

The connections between the nasal cartilages weaken, leading to ptosis of the wings of the nose and result in its pyriform appearance. Combined with the resorption of the jaw, this leads to the accentuation of the nasolabial groove [38].

Resorption of the mandible and ptosis of the chin lead to the relief of the underlying structures, even the submandibular gland. Ptosis of the chin fat, excess skin, and the facial portion of the platysma creates the appearance of a turkey neck. The additional contraction of the platysma, in an attempt to create a support for the floor of the mouth and the deep structures of the throat, forms vertical bands at this level. The progressive descent of the hyoid and the larynx leads to a decrease in the cervicomental angle [37].

All these changes that occur gradually can lead, for an experienced eye, to an accurate estimate of the age of the examined subject.

MRI offers the most definitive visualization of cervicofacial soft tissues, whereas MRAs utilize MRI principles and techniques to concentrate specifically on blood vessel imaging. It is capable of depicting detailed stratigraphic anatomical information, yielding images akin to those from CT scans but with superior soft tissue differentiation. 

**Figure 4:**
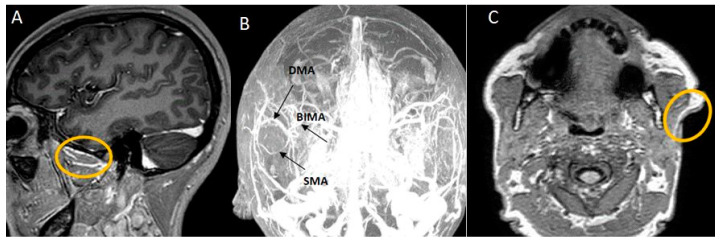
3T MRA imaging blood sources of masseteric regions. (**A**) SMAS masseteric branches from the internal maxilary artery; (**B**) Masseteric main arterial anasthomoses; (**C**) The auricular artery’s parotid branch.

A recent study using a 3.0T MRA device was conducted on proper imaging parameters using TR: 3000–4500 ms/TE: 80–150 ms for T2 weighted sequences and TR: 600–650 ms/TE: 15–25 ms for T1 weighted sequences with a 3 mm slice thickness and 20% interslice gap. The image analyses were made with a Dicom Viewer unit. Using this method, the authors identified the main source vessels of the regions explored by histological and IHC techniques and superimposed the results with the topographical and morphological aspects from the histological and IHC tests (Figure 4) [32].

### 2.4. The Internal (Structural) Changes of Face Aging

The rapid and explosive development of the computing technique in the last two decades has led to a revitalization of morpho-histological, immunohistochemical, molecular, and radiological studies.

When considering facial aging, we typically focus on the soft tissue, skin, and fat that deteriorate and become bigger and flabbier, and generally we just lift everything back up and remove some skin to keep it tight in place. The ideal solution would be to combine the traditional lifting with fat injections to add volume to the face [39].

The changes are the result of progressive gravity-related migration of facial tissue, particularly adipose tissue. In fact, there is laxity and sagging of the fascia, of the existing muscles and ligaments, phenomena that lead to the loss of facial volume at the level of the cheeks and their falling towards the mandible, together with a bilateral skin excess. The cartilaginous skeleton of the nose weakens and elongates resulting in ptosis of the nasal lobe, lengthening of the nose, and occasionally increasing the possibilities of nasal obstruction. In addition to the gradual increase in skin laxity, depletion of subcutaneous fat, and resorption of alveolar bone, an apparent surplus of skin and soft tissue accumulates in the lower face and temporal region. The facial skeleton loses in height (at the level of the maxilla and mandible) and gains horizontally and in depth.

These events determine the downward aspect of the chin, the loss of the clear boundary between the submandibular region and the neck, as well as the characteristic “turkey neck” appearance. As you get older, the hyoid bone and the larynx descend, so that the middle and lower part of the neck are more prominent. The anterior edge of the platysma muscle loses tone and separates, resulting in the lamellar appearance of the aged neck. Submental fat can prolapse between these blades, further leading to loss of the cervicomental angle. Some patients develop a relative downward projection of the zygomatic eminence or the chin due to redistribution of skin, soft tissues, and subcutaneous fat. As the soft tissues in the central part of the face descend inferiorly, laterally, and anteriorly, the nasolabial fold becomes more pronounced, especially at rest. The sliding of the cheeks, as well as the progressive absorption of the subcutaneous adipose tissue around the mouth opening, determines an accentuated definition of the nasolabial fold. Strong perioral tissue also resorbs, especially in edentulous patients, causing excess skin and soft tissue at this level.

## 3. Anatomical Base of Modern Surgery—Superficial Musculoaponeurotic System

The discovery of the SMAS (superficial cervicofacial musculoaponeurotic system) in 1974 wrote a new and crucial page in cervicofacial anatomy, and plastic and reconstructive surgery. At the same time, this discovery lays the foundation stone in a long line of research studies of SMAS [40]. Morphological and functional understanding of age-related changes in SMAS is the basis for choosing the appropriate technique in surgical procedures or in medical aesthetic and reconstructive procedures.

Anatomical studies using the operating microscope, combined with morphohistochemical analysis of the complexity of the muscles and connective tissue comprising the SMAS, has resulted in innovative discoveries in the field of tissue bioengineering [41,42].

A relatively recent direction of research, which is increasingly gaining momentum, is the description of the superficial and deep fat compartments of the face. Starting with the research done by Rohrich and Pessa [43,44,45], and its radiological confirmation by Gierloff (2012), further support was provided for the theory of facial deflation and volume changes in these compartments over time [46,47]. Defining the anatomical limits of these fat compartments (nasolabial, medial, middle, superficial lateral, deep medial cheek, suborbicularis, buccal, and periorbital) brings a topographical and clear view of the superficial cervicofacial soft tissues. The same researchers pioneered filler injections, thus highlighting the major topographical changes that occur with limited volumetric changes in these specific areas of the face.

Recent discoveries in the anatomy of the facial fat compartment have conceptually and interventionally revolutionized the approach to adding volume to specific deflated soft tissue compartments, creating a more individualized youthful restoration of the face [48,49]. Rendering or even reconstructing the characteristics of facial youth starts from the skeletal structure and goes progressively up to the SMAS. Improvements in midface skeletal proportions through the use of calcium hydroxyapatite or implants, restoration of proper soft tissue position and volume via fat grafting, and manipulation of the SMAS and palpebral structures, as well as botulinum toxin injections or resurfacing, allow for the restoration of youthful facial features in a meticulously tailored manner, addressing individual-specific changes gradually and systematically. In this sense, the modern techniques of 3D scanning of the face, correlated with the 3D printing of tissue supports, intervene in the techniques of reconstruction of the facial bones.

All changes in the shape of the facial skeleton, soft tissue, retaining ligaments, fat compartments, and skin envelope plays a significant role in facial aging to varying degrees.

Complex IHC studies, correlated with high-performance imaging studies reveal both new data about the morphofunctional aspects of the soft, superficial layers of the face as well as their changes that appear with age [48].

CR studies continued to develop throughout the 20th century. A 2020 study fed rats with fewer calories than their ad-libitum counterparts and found that 57% of the age-related genetic changes that occurred in normal rats did not occur in calorie-restricted rats [49]. MRI or CT imaging of SMAS are also evolving to demask its implications into the phenomena of the aging face [50,51,52].

A 2021 study of *C. elegans* worms discovered the tumor suppressor phosphatase and protein tension homolog (PTEN) [53]. PTEN is a tumor suppressor, which means it helps prevent the cell from engaging in any unhealthy behavior. It contains a single-chain amino acid that supports health and longevity, while counteracting other naturally declining biological changes. Such studies offer the promise of genetic modification that can help keep organisms healthy while promoting longevity. These studies investigate the background of chronic inflammatory disease related to obesity and also to aging phenomena [54].

## 4. Perspectives

**Figure 5:**
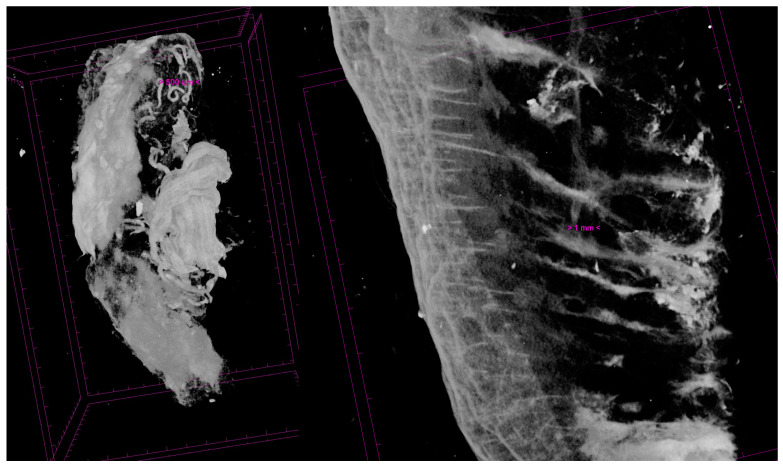
MicroCT relevance of intraSMAS vascular plexus (**left**) and collagen fibers from SMAS attaching onto the skin (**right**) (personal collection).

Electron microscopy, special IHC techniques, and even microCT show extremely promising results in the study of aging phenomena through quantitative and qualitative determinations of the constituents of the superficial soft layers of this region (Figure 5) [32]. Confocal immunofluorescence with specific vascular and collagen markers, as well as electron microscopy studies, are increasingly important in aging face research. 

Collagen is a major component of the extracellular matrix that becomes fragmented and coarsely distributed over time. Thus, its total amount decreases due to increased activity of matrix metalloproteinases and impaired transforming of growth factor-β signaling induced by reactive oxygen species generated during aging [55]. These phenomena prevent the mechanical interaction between fibroblasts and the extracellular matrix and lead to the deterioration of fibroblast function and further decrease in the amount of dermal collagen. There are other components of the extracellular matrix that also change during aging, such as elastic fibers, glycosaminoglycans (GAGs), and proteoglycans (PGs). Elastic fibers are thus quantitatively reduced in intrinsically aged skin, but accumulate abnormally in photoaged skin. The appearance of the clinical characteristics of aging is the result of the reduction in the levels of the mentioned functional dermal components [56]. Previous studies have reported conflicting results regarding the aging molecular mechanism considering GAGs and PGs. Future research will have to go deeper into this major topic to increase our knowledge. 

By combining quantitative data analysis with qualitative insights from interviews, surveys, and observational studies, researchers can develop a more nuanced understanding of aging and its impact on individuals and society. With such an evolution and, with a revolution in the field of scientific research techniques, we can hope that we will finally find the much-coveted fountain of youth.

## Data Availability

Data are contained within the article.

## References

[1] Rowe J.W., Carr D.C. (2018). Successful Aging: History and Prospects.

[2] Bjork C. (2021). Plato, Xenophon, and the Uneven Temporalities of Ethos in the Trial of Socrates. Philos. Rhetor..

[3] Stambler I. (2020). History of Life-Extensionism. Encyclopedia of Biomedical Gerontology.

[4] Vos E.E., de Bruin S.R., van der Beek A.J., Proper K.I. (2021). “It’s Like Juggling, Constantly Trying to Keep All Balls in the Air”: A Qualitative Study of the Support Needs of Working Caregivers Taking Care of Older Adults. Int. J. Environ. Res. Public Health.

[5] Nagaratnam N., Nagaratnam N., Nagaratnam K., Cheuk G. (2019). Ageing and Longevity. Advanced Age Geriatric Care: A Comprehensive Guide.

[6] Kyriazis M. (2020). Ageing Throughout History: The Evolution of Human Lifespan. J. Mol. Evol..

[7] Ames D. (2017). Ageing: A Very Short Introduction Nancy A. Pachana Oxford: Oxford University Press, 2016, £7.99/$US11.99, paperback, 132 pp. ISBN 978-0-19-872532-9. Int. Psychogeriatr..

[8] Rodrigues C.E., Grandt C.L., Alwafa R.A., Badrasawi M., Aleksandrova K. (2023). Determinants and indicators of successful aging as a multidimensional outcome: A systematic review of longitudinal studies. Front. Public Health.

[9] Rohrich R.J., Avashia Y.J., Savetsky I.L. (2021). Prediction of Facial Aging Using the Facial Fat Compartments. Plast. Reconstr. Surg..

[10] Coleman S.R., Grover R. (2006). The anatomy of the aging face: Volume loss and changes in 3-dimensional topography. Aesthet. Surg. J..

[11] Park D.M. (2015). Total facelift: Forehead lift, midface lift, and neck lift. Arch. Plast. Surg..

[12] Hebel N.S.D., Boonipat T., Lin J., Shapiro D., Bite U. (2023). Artificial Intelligence in Surgical Evaluation: A Study of Facial Rejuvenation Techniques. Aesthet. Surg. J. Open Forum.

[13] Murphrey M.B., Miao J.H., Zito P.M. (2024). Histology, Stratum Corneum. StatPearls.

[14] Cornaro L. (1979). The Art of Living Long.

[15] Rush B. (1797). Medical Inquiries and Observations.

[16] Cole T.R. (1992). The Journey of Life: A Cultural History of Aging in America.

[17] Mudunuri A., Chandrakanth M., Khan S., Sura C., Kumar N., Tung S. (2024). Diet-induced plasticity of life-history traits and gene expression in outbred Drosophila melanogaster population. Ecol. Evol..

[18] Bellini F., Lancaster J.A.T., Raiswell R. (2018). Diet and Hygiene Between Ethics and Medicine: Evidence and the Reception of Alvise Cornaro’s La Vita Sobria in Early Seventeenth-Century England. Evidence in the Age of the New Sciences.

[19] Sen P., Shah P.P., Nativio R., Berger S.L. (2016). Epigenetic Mechanisms of Longevity and Aging. Cell.

[20] Zhang S., Li F., Zhou T., Wang G., Li Z. (2020). Caenorhabditis elegans as a Useful Model for Studying Aging Mutations. Front. Endocrinol..

[21] Hull B., Irby I.M., Miller K.M., Anderson A., Gardea E.A., Sutphin G.L. (2024). Experimental variables that impact outcomes in Caenorhabditis elegans aging stress response. bioRxiv.

[22] Klass M.R. (1977). Aging in the nematode Caenorhabditis elegans: Major biological and environmental factors influencing life span. Mech. Ageing Dev..

[23] Mey G.M., Mey J.T. (2022). Emerging Nutrition Approaches to Support the Mind and Muscle for Healthy Aging. Recent. Prog. Nutr..

[24] Martin G.M., LaMarco K., Strauss E., Kelner L.K. (2003). Research on aging: The end of the beginning. Science.

[25] Goldsmith T.C., Gu D., Dupre M.E. (2019). Timeline of Aging Research. Encyclopedia of Gerontology and Population Aging.

[26] Chaudhary M., Khan A., Gupta M. (2020). Skin Ageing: Pathophysiology and Current Market Treatment Approaches. Curr. Aging Sci..

[27] Kuruoglu D., Salinas C.A., Kirk D.S., Wong C.H., Sharaf B.A. (2023). Brow and Eyelid Rejuvenation: Trends from the 100 Most Cited Articles over 30 Years. Medicina.

[28] Gallego-Jara J., Ortega Á., Lozano Terol G., Sola Martínez R.A., Cánovas Díaz M., de Diego Puente T. (2021). Bacterial Sirtuins Overview: An Open Niche to Explore. Front. Microbiol..

[29] Pu K., Feng Y., Tang Q., Yang G., Xu C. (2024). Review of dietary patterns and gastric cancer risk: Epidemiology and biological evidence. Front. Oncol..

[30] Clarfield A.M. (2007). Geriatrics: The Diseases of Old Age and Their Treatment. BMJ.

[31] Frâncu L.L., Hînganu D., Hînganu M.V. (2013). Anatomical evidence regarding the existence of sustentaculum facies. Rom. J. Morphol. Embryol..

[32] Cucu R.P., Hînganu M.V., Costan V.V., Lozneanu L., Boişteanu O., Tamaş C., Negru D., Hînganu D. (2024). Morphofunctional and histological patterns of blood vessels in the superficial cervicofacial musculoaponeurotic system in midlateral face regions. Ann. Anat..

[33] Lowry W.E. (2020). Its written all over your face: The molecular and physiological consequences of aging skin. Mech. Ageing Dev..

[34] Christian L.M., Wilson S.J., Madison A.A., Prakash R.S., Burd C.E., Rosko A.E., Kiecolt-Glaser J.K. (2023). Understanding the health effects of caregiving stress: New directions in molecular aging. Ageing Res. Rev..

[35] Coleman S.R. (2006). Structural fat grafting: More than a permanent filler. Plast. Reconstr. Surg..

[36] Gosain A.K., Klein M.H., Sudhakar P.V., Prost R.W. (2005). A volumetric analysis of soft-tissue changes in the aging midface using high-resolution MRI: Implications for facial rejuvenation. Plast. Reconstr. Surg..

[37] Flanagan E.W., Most J., Mey J.T., Redman L.M. (2020). Calorie Restriction and Aging in Humans. Annu. Rev. Nutr..

[38] Pessa J.E., Zadoo V.P., Yuan C., Ayedelotte J.D., Cuellar F.J., Cochran C.S., Mutimer K.L., Garza J.R. (1999). Concertina effect and facial aging: Nonlinear aspects of youthfulness and skeletal remodeling, and why, perhaps, infants have jowls. Plast. Reconstr. Surg..

[39] Cotofana S., Fratila A.A., Schenck T.L., Redka-Swoboda W., Zilinsky I., Pavicic T. (2016). The Anatomy of the Aging Face: A Review. Facial Plast. Surg..

[40] Tessier P. (1989). [Subperiosteal face-lift]. Ann. Chir. Plast. Esthet..

[41] Ghassemi A., Prescher A., Riediger D., Axer H. (2003). Anatomy of the SMAS revisited. Aesthetic Plast. Surg..

[42] Farkas J.P., Pessa J.E., Hubbard B., Rohrich R.J. (2013). The Science and Theory behind Facial Aging. Plast. Reconstr. Surg. Glob. Open.

[43] Rohrich R.J., Pessa J.E., Ristow B. (2008). The youthful cheek and the deep medial fat compartment. Plast. Reconstr. Surg..

[44] Trévidic P., Kaufman-Janette J., Weinkle S., Wu R., Dhillon B., Antunes S., Macé E., Maffert P. (2022). Injection Guidelines for Treating Midface Volume Deficiency with Hyaluronic Acid Fillers: The ATP Approach (Anatomy, Techniques, Products). Aesthet. Surg. J..

[45] Kim C.H. (2022). Evaluating the Compartment-Specific Effects in Superficial Facial Fat Compartments after Thread-Lifts by the Tensiometer and FACE-Q. Aesthet. Surg. J. Open Forum.

[46] Gierloff M., Stöhring C., Buder T., Gassling V., Açil Y., Wiltfang J. (2012). Aging changes of the midfacial fat compartments: A computed tomographic study. Plast. Reconstr. Surg..

[47] Sarigul Guduk S., Cevik Cenkeri H., Derin Cicek E., Kus S. (2022). Evaluation of aging changes of the superficial fat compartments of the midface over time: A computed tomography study. J. Cosmet. Dermatol..

[48] Bay E.Y., Topal I.O. (2023). Aging Skin and Anti-Aging Strategies. Explor. Res. Hypothesis Med..

[49] Zhai J., Kongsberg W.H., Pan Y., Hao C., Wang X., Sun J. (2023). Caloric restriction induced epigenetic effects on aging. Front. Cell Dev. Biol..

[50] Okuda I., Akita K., Komemushi T., Irimoto M., Nakajima Y. (2021). Basic Consideration for Facial Aging: Analyses of the Superficial Musculoaponeurotic System Based on Anatomy. Aesthet. Surg. J..

[51] Okuda I., Abe K., Yoshioka N., Komemushi T., Jinzaki M., Ohjimi H. (2023). Objective Analysis of Age-Related Changes in the Superficial Musculoaponeurotic System in Japanese Females Using Computed Tomography. Aesthet. Surg. J. Open Forum.

[52] Hînganu M.V., Hînganu D., Palade O.D., Eva I., Volovăţ S.R., Cucu R.P., Costan V.V. (2023). Clinical and morphofunctional identity of the nasal SMAS. Rom. J. Morphol. Embryol..

[53] Katsanos D., Ferrando-Marco M., Razzaq I., Aughey G., Southall T.D., Barkoulas M. (2021). Gene expression profiling of epidermal cell types in C. elegans using Targeted DamID. Development.

[54] Li X., Li C., Zhang W., Wang Y., Qian P., Huang H. (2023). Inflammation and aging: Signaling pathways and intervention therapies. Signal Transduct. Target. Ther..

[55] Shin J.W., Kwon S.H., Choi J.Y., Na J.I., Huh C.H., Choi H.R., Park K.C. (2019). Molecular Mechanisms of Dermal Aging and Antiaging Approaches. Int. J. Mol. Sci..

[56] He X., Gao X., Xie W. (2023). Research Progress in Skin Aging, Metabolism, and Related Products. Int. J. Mol. Sci..

